# Stem Cell and Tissue Engineering Applications in Orthopaedics and Musculoskeletal Medicine

**DOI:** 10.1155/2012/403170

**Published:** 2012-03-25

**Authors:** Wasim S. Khan, Umile Giuseppe Longo, Adetola Adesida, Vincenzo Denaro

**Affiliations:** ^1^UCL Division of Surgery, Academic Orthopaedic Registrar, RNOH Rotation, Stanmore, London HA7 4LP, UK; ^2^Department of Orthopaedic and Trauma Surgery, Campus Bio-Medico University, Rome, Italy; ^3^Faculty of Medicine and Dentistry, University of Alberta, Edmonton, AB, Canada T6G 2R7

Over the past two decades, there has been a tremendous interest in the application of tissue engineering protocols and stem cells, particularly, mesenchymal stem cells (MSCs) to develop cell-based strategies for regeneration, replacement, or repair of damaged tissues. The 1990s brought the advent of tissue engineering, where MSCs were differentiated in the presence of specific bioactive molecules towards bone, cartilage and other musculoskeletal tissues. The beginning of the 21st century saw the emergence of cell-based clinical therapies using MSCs at three different approaches. Firstly, tissue-engineering approaches in which MSCs are seeded into three-dimensional (3-D) scaffolds in order to generate functional tissues for replacement of defective tissues. Secondly, we see the use of MSC transplantation to replace defective host cells and thirdly, harnessing the properties of MSCs to act as cytokine/growth factor producers to stimulate repair or inhibit degenerative processes. 

Reflecting the enormous interest of both our readership and authors in this promising field, we are pleased to present this special issue. This special issue presents a total of 21 paper including research papers and comprehensive reviews that aim to provide a better understanding of current basic research findings, clinical data, and the challenges encountered on the application of biologicals (cytokines/growth factors), tissue engineering, and stem cells in the treatment of musculoskeletal lesions affecting bone, cartilage, intervertebral discs, ligaments, menisci, muscle, and tendons.

The treatment of large bone defects remains a major challenge for orthopaedic reconstructive surgeons. Generation of bone grafts with osteoconductive, osteoinductive, and osteogenic properties via tissue engineering strategies may resolve this problem. W. Khan et al. review available literature on bone tissue engineering to understand its principle, current limitations, and clinical relevance in bone reconstructive surgery.

Hyaline cartilage is an avascular tissue covering the end of long bones in diarthrodial joints where it fosters near frictionless movement and load bearing. Current treatment options for damaged hyaline cartilage lead to the formation of fibrocartilage tissue that neither recapitulates the molecular phenotype nor the biomechanical properties of hyaline cartilage. While MSCs have the capacity to be differentiated into cartilage cells: chondrocytes, current methods of chondrogenic induction of MSCs result in fibrocartilage formation and expression of templates for osteogenesis. In this special issue, U. Longo et al. provide a comprehensive review of available literature on the role of gene therapy and MSCs to develop therapeutic strategies for hyaline cartilage regeneration.

Low back pain is one of the largest health problems in the Western world today affecting 8 in 10 of the adult population at some point in their lives and has an enormous impact on Western economies due to occupational incapacities and healthcare cost. While the causes of low-back pain are thought to be multifactorial, the degeneration of intervertebral discs (IVD) is frequently seen in almost all cases. U. Longo et al. provide two review papers on the prevention and management of IVD degeneration. One paper focused on the role of biologics: growth factors and anticatabolic agents, to stimulate synthesis of extracellular matrix molecules that are central to IVD functionality and inhibition of the enzymes responsible for IVD degradation. The other paper provided a review of *in vitro* and *in vivo* evidence on the potential application of MSCs to treat IVD degeneration. 

Ligaments possess the remarkable capacity to connect two or more bones, stabilize joints, and to sustain excessive mechanical loads. However, they have a limited reparative and regenerative capacity. Current treatment modalities, that comprise the use of autografts and allografts, suffer from a variety of issues that include donor site morbidity, pain, altered harvest biomechanics, and disease transmission. Hence there is a need for new treatments. Tissue engineering strategies have been advocated as a potential approach to resolve these problems. Calgor Yilgor et al. provide a review paper summarizing the application of biologics, cells (MSCs and fibroblast) scaffolds and bioreactors in ligament regeneration. While in a paper, W. Yates et al. focused their review of current literature on the relevance of ligament tissue engineering on anterior cruciate ligament reconstruction. S. Rathbone et al. report that most British orthopaedic surgeons will use tissue-engineered anterior cruciate ligament in clinical practice.

The meniscus is a vital tissue for normal healthy knee joint biomechanics. Meniscus injury is a major risk factor for the development of osteoarthritis. Unfortunately, the reparative capacity of the meniscus is limited to its vascularized region. In a research article, D. Ferris et al. used equine autologous bone marrow stromal mesenchymal stem cell and fibrin glue to demonstrate healing of lacerated equine menisci in a nude mice model. Their study may serve as a promising model system to study cell-based therapies for meniscus repair. In their paper, U. Longo et al. produced a review of available literature on current strategies of tissue engineering for the management of meniscal defects. In another paper, U. Longo et al. focused their review on proposed biological strategies directed to enhance meniscus repair in the avascular region.

Despite the fact that skeletal muscle has the capacity to repair and regenerate, muscle function is compromised due fibrotic scar tissue formation after injury. U. Longo et al. review current literature on the application of biologicals, such as platelet rich plasma and tissue engineering strategies comprising scaffolds and MSCs in the management of skeletal muscle injuries. While S. MacLean et al. discuss current literature on different stem cell types, gene therapy, and tissue engineering approaches that may be applicable to skeletal muscle regeneration following injury and muscular dystrophy.

Tendons are central to normal functionality of the musculoskeletal system where they connect muscle and bone to produce joint movement. Unfortunately, the reparative and regenerative capacities of tendons are limited because of their low cellularity. Tendon injuries are a common cause of morbidity in the population and account for a significant health care burden. Preclinical studies with a variety of cell types including MSCs, tendocytes, dermal fibroblast, have been evaluated for tendon repair or regeneration. While these studies show promise of cell-based options in treating tendon disorder, there have been little clinical applications. U. Young reviews the application of stem cells in tendon disorders. L. Gulotta et al. review current literature on the use of stem cells with or without growth factors to enhance tendon repair. S. Maclean et al. review stem cell-based tissue engineering strategies in the management of tendon injuries. In a clinical study, C. Pascual-Garrido et al. report a 5-year follow up clinical improvement in a small cohort of patients with chronic patellar tendinopathy after treatment with untreated bone marrow stromal stem cells. N. Maffulli et al. discuss current literature on tissue engineering approaches to treat rotator cuff tears. While U. Longo et al. review current literature on available biological and synthetic scaffolds for tendon tissue engineering.

The study of stem cells isolated from a variety of tissues often offers unique insights into similarities or differences in differentiation potential between stem cell populations. Schmitt and coauthors discuss current literature on different stem cells use in orthopaedic regenerative medicine. While in a research paper, Marmotti and colleagues report a novel method of isolating stem cells from umbilical cord for orthopaedic tissue engineering. Furthermore, they showed evidence that the stem cells had the capacity to undergo myogenesis, adipogenesis, chondrogenesis, and osteogenesis. E. Fossett and W. Khan discuss in a review how MSCs seeding density, age, and gender of MSCs donor affect the proliferative capacity of MSCs.

In summary, we are very excited to present to our readers this special collection of outstanding review and research articles and would like to take this opportunity to thank all our authors for their contributions and support to *Stem Cells International*.

*Wasim S. Khan* *Wasim S. Khan* *Umile Giuseppe Longo* *Umile Giuseppe Longo* *Adetola Adesida* *Adetola Adesida* *Vincenzo Denaro* *Vincenzo Denaro*

